# Prioritising recommendations following analyses of adverse events in healthcare: a systematic review

**DOI:** 10.1136/bmjoq-2019-000843

**Published:** 2020-10-09

**Authors:** Kelly Bos, Maarten J van der Laan, Dave A Dongelmans

**Affiliations:** 1 Department of Surgery, Amsterdam UMC - location AMC, Amsterdam, the Netherlands; 2 Department of Surgery, University Medical Centre Groningen, Groningen, the Netherlands; 3 Department of Intensive Care Medicine, Amsterdam UMC - location AMC, Amsterdam, the Netherlands

**Keywords:** adverse events, epidemiology and detection, incident reporting, patient safety, quality improvement, root cause analysis

## Abstract

**Purpose:**

The purpose of this systematic review was to identify an appropriate method—a user-friendly and validated method—that prioritises recommendations following analyses of adverse events (AEs) based on objective features.

**Data sources:**

The electronic databases PubMed/MEDLINE, Embase (Ovid), Cochrane Library, PsycINFO (Ovid) and ERIC (Ovid) were searched.

**Study selection:**

Studies were considered eligible when reporting on methods to prioritise recommendations.

**Data extraction:**

Two teams of reviewers performed the data extraction which was defined prior to this phase.

**Results of data synthesis:**

Eleven methods were identified that are designed to prioritise recommendations. After completing the data extraction, none of the methods met all the predefined criteria. Nine methods were considered user-friendly. One study validated the developed method. Five methods prioritised recommendations based on objective features, not affected by personal opinion or knowledge and expected to be reproducible by different users.

**Conclusion:**

There are several methods available to prioritise recommendations following analyses of AEs. All these methods can be used to discuss and select recommendations for implementation. None of the methods is a user-friendly and validated method that prioritises recommendations based on objective features. Although there are possibilities to further improve their features, the ‘Typology of safety functions’ by de Dianous and Fiévez, and the ‘Hierarchy of hazard controls’ by McCaughan have the most potential to select high-quality recommendations as they have only a few clearly defined categories in a well-arranged ordinal sequence.

## Introduction

Adverse events (AEs)—defined as unexpected occurrences involving death or serious physical or psychological injury—affect numerous patients in healthcare organisations worldwide.^1^ Solely in the Netherlands, 1272 AEs were reported by hospitals, private clinics and rehabilitation centres in 2016.^2^ Many countries have developed a system to register and analyse these AEs in an attempt to prevent recurrence and improve patient safety.^3^ However, this has not yet resulted in a decrease in the number of AEs.^4^ Although this suggests that learning from AEs is insufficient, the number of AEs alone is not a measure for the learning effect. An increase in the number of AEs does not necessarily mean healthcare has become less safe. AEs could be better recognised and, therefore, reported more frequently. Recurrence of similar AEs is a better measure for the effect of learning from AEs.

In the Netherlands, 60 cases of wrong-site surgery were reported to the Dutch Healthcare Inspectorate between 2014 and 2016.^5^ Between April 2014 and March 2015, 124 cases of wrong-site surgery were reported in the UK; and in the USA these events occur approximately 1300 to 2700 times annually.^6 7^ Despite previous analyses of these type of AEs, they still recur on a daily basis worldwide. Recurrence of similar AEs strongly suggests learning from AEs is complex and unsatisfactory. This might be partly due to the quality of recommendations following analyses of AEs. In Australia, the quality of recommendations following analyses of AEs was recently assessed to investigate their effectiveness and sustainability. Of all the 1137 recommendations evaluated, only 8% were of high-quality.^8^ In order to achieve a potential reduction in (similar) AEs in healthcare, it seems plausible to focus on implementing the high-quality recommendations.

Insight in the basic conditions of a high-quality recommendation will improve the quality of the recommendations and ultimately improve patient safety. Furthermore, it might aid in directing time and resources when selecting recommendations for implementation. Therefore, the purpose of this systematic review was to identify an appropriate method—a user-friendly, validated method—that prioritises recommendations following analyses of AEs in healthcare based on objective features.

## Methods

This review protocol was registered in PROSPERO, the international prospective register of systematic reviews (registration number CRD42018092002) and reported according to the Preferred Reporting Items for Systematic reviews and Meta-Analyses (PRISMA) statement.^9 10^

### Search strategy

The electronic databases PubMed/MEDLINE, Embase (Ovid), Cochrane Library, PsycINFO (Ovid) and ERIC (Ovid) were searched for published studies on recommendations following analyses of AEs with the assistance of a clinical librarian on 9 March 2018. Search terms included: healthcare, hospital, quality improvement, safety management, recommendation, safety intervention, remedial action, improvement tool, usefulness and usability. The detailed search strategies are presented in online supplemental file 1. No restrictions regarding language, study design or publication date were applied. The reference lists of eligible studies were manually screened to identify additional relevant studies. Through a human factor consultant and engineer at Intergo Human Factors and Ergonomics (Acknowledgements), both specialised in dealing with AEs in safety-critical industries, eligible studies from other industries than healthcare were obtained.

10.1136/bmjoq-2019-000843.supp1 Supplementary data

### Study selection

Data was processed using the Covidence systematic review software (Veritas Health Innovation, Melbourne, Australia; available at www.covidence.org). Prior to the study selection, duplicates were removed. Studies were considered eligible when reporting on methods to prioritise recommendations following analyses of AEs. The methods must be intended for or applicable to healthcare. Title and abstract screening was performed by two teams of reviewers (KB/DAD or KB/MJvdL). In case of disagreement, consensus was reached through discussion within the teams, and when necessary, the opinion of the third reviewer was obtained. If the full-text study could not be extracted, the corresponding author of the concerning study was contacted and the full text was requested. Of all selected studies, the full text was analysed by both teams (KB/DAD or KB/MJvdL) and all reviewers agreed on the final selection of studies.

### Data extraction

Two teams of reviewers (KB/DAD or KB/MJvdL) performed the data extraction that was defined by the authors prior to this phase. In case of different outcomes, consensus was reached through discussion.

### Definition of outcomes

Predefined criteria to assess the quality of the method prioritising recommendations were used to extract the data. The primary outcomes were:

- ‘user-friendly’: easy to understand, not time consuming and with simple calculations;
- ‘validation’: the method is tested and results are considered to be reproducible when used different times by the same user and/or by different users;
- ‘prioritisation of recommendations only’: recommendations can be prioritised without taking other factors such as implementation or other steps of incident analysis into consideration;
- ‘prioritisation based on objective features’: features or scores were considered to be objective when personal opinion or knowledge was not expected to affect the scoring process, and the scoring could be expected to be similar when performed by different users.

Other outcomes were the ‘description of the method’, ‘categories or scores used for prioritisation’ and ‘development of the method’. This included what the method was based on, which experts were involved in the development and ‘description of the validation’. ‘Year’, ‘country’ and ‘industry’ of publication and ‘applicability to healthcare’ were also extracted. Since the data was not appropriate for quantitative synthesis, no additional statistical analyses were performed.

### Quality assessment

Unfortunately, no appropriate quality assessment tool was available for this type of research. The available quality assessment tools for qualitative research do not apply to all types of qualitative research especially when no experimental data, cohorts or interventions were evaluated.

### Patient and public Involvement

Patients were not involved in this study.

## Results

### Included studies

**Figure 1:**
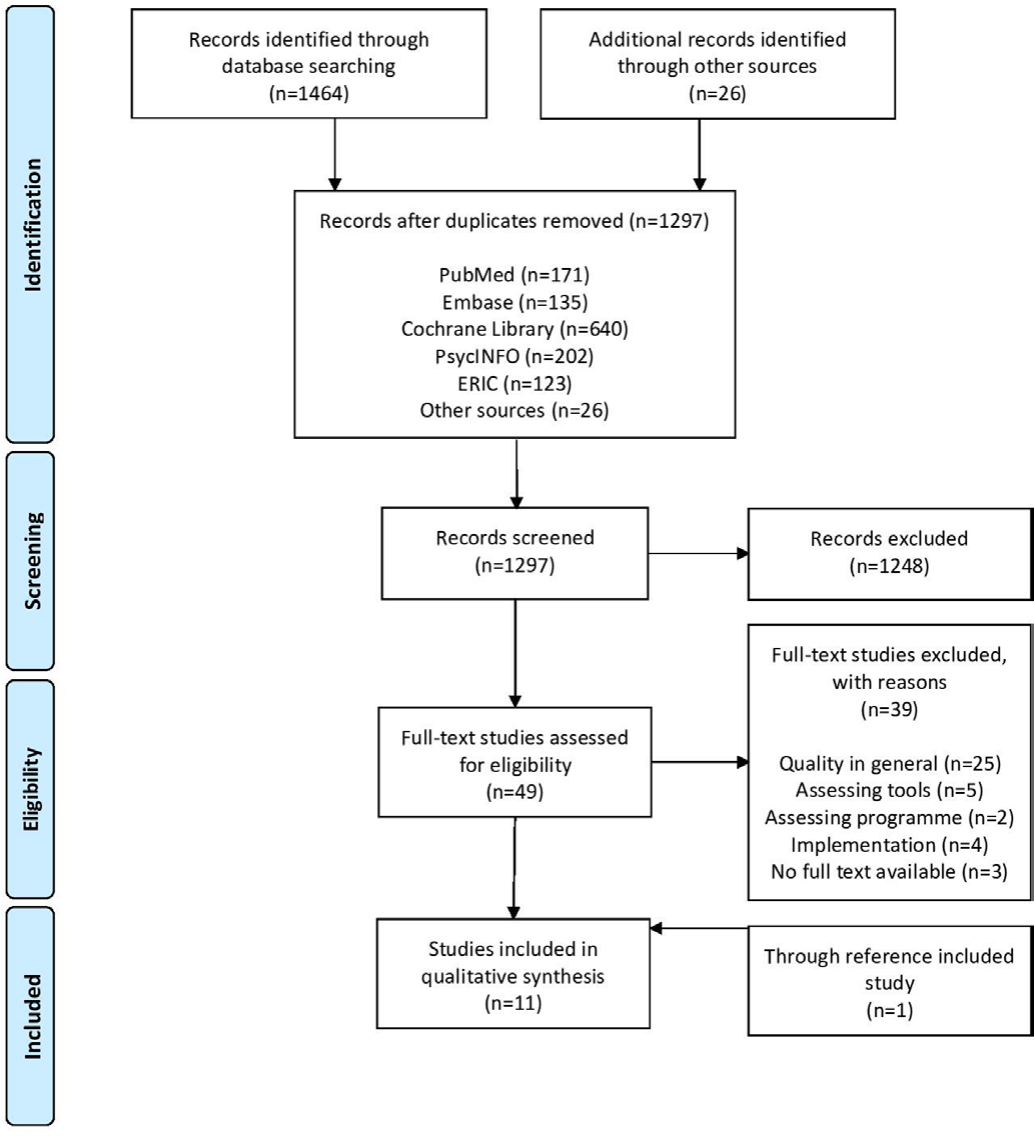
PRISMA flow diagram. PRISMA Preferred Reporting Items for Systematic Reviews and Meta-Analyses.

The systematic search identified a total of 1297 studies. After screening of title and abstract, 49 studies were considered eligible. After reviewing the full-text paper, 10 studies were included. One study was added to the included studies after screening the references of the included studies. More detailed information regarding the study selection process is presented in figure 1.

### Study characteristics

The 11 included studies were published between 1990 and 2017.^11–21^ The studies originated from the USA, Norway, Spain, Ireland, the UK, Turkey, France and Belgium. The methods of eight studies were intended for healthcare.^11 12 14 16 17 19–21^ Three studies were intended for safety-critical industries in general^13 15 18^ and two of these were applicable to healthcare as stated by the authors.^15 18^ The study by de Dianous and Fiévez did not explicitly state the applicability to healthcare.^13^ However, due to the generalisability of the described methodology, the reviewers decided the described method was applicable to healthcare.

### Method specifics

All the 11 studies described a method to prioritise recommendations following analyses of AEs, therefore differentiating between high-quality and low-quality recommendations. Four studies used categories^13 16–18^ and seven studies used ranking numbers to accomplish this.^11 12 14 15 19–21^ Nine methods were reviewed as user-friendly by the reviewers.^11–19^ The two methods not considered user-friendly were those that used extensive calculations in order to prioritise the recommendations.^20 21^ Specifics regarding the methods and their categories can be found in table 1.

**Table 1:** Method characteristics and prioritisation of recommendations per method

Study	Name of method	Description of method and prioritisation of recommendations	Categories used for prioritisation from poor to excellent
Brandrud *et al*^11^	Change Process and Outcome evaluation instrument Scale	The scale comprises 20 items, of which six items address recommendations	The items addressing recommendations were rated on a 1 to 5 scale
Coburn *et al*^12^	NR	For each recommendation, four criteria were rated	The criteria were rated on a 1 to 5 scale
de Dianous and Fiévez^13^	Typology of safety functions	Recommendations are placed in one of four categories, according to their intended effect	‘Limit, reduce or mitigate’, ‘control’, ‘prevent’, ‘avoid’
Flottorp *et al*^14^	Tailored Implementation for Chronic Diseases’ checklist. Worksheet 1: prioritisation of recommendations	The worksheet addresses three criteria for recommendations	The criteria are rated on a 1 to 5 scale for each recommendation
Geller *et al*^15^	Taxonomy of behaviour change strategies to guide intervention development and evaluation	Each recommendation is assigned one or more of 24 behaviour change techniques	The sum of points per behaviour change technique will prioritise the recommendation for each specific technique: 1 to 4
Hettinger *et al*^16^	Model of sustainability and effectiveness in root cause analysis solutions	Each recommendation is placed in one of 13 solution categories in which they intend to intervene, which were placed on a two-dimensional framework	Effectiveness (y-axis): Minimal—low—moderate—high Sustainability (x-axis): Minimal—low—moderate—high
McCaughan^17^	Hierarchy of hazard controls	Recommendations are placed in one of five categories in which they intend to intervene or according to their intended effect	Work practice controls, administrative procedures, engineering controls, substitution and elimination
McLeod *et al*^18^	Summary of the relationships between components of a barrier system	Recommendations are placed in one of four categories in which they intend to intervene or according to their intended effect	Human—operational, human—organisational, combination and technical
Mira *et al*^19^	NR	Recommendations are assessed for understandability, feasibility and usefulness	The items are rated using a scale of 0 to 10
Rodriguez-Gonzalez *et al*^20^	NR	Recommendations are prioritised based on the order in which they should be implemented by calculating a risk priority number	Priority of implementation on 5 to 1
Testik *et al*^21^	Analytical Hierarchy Process methodology	A multicriteria decision-making method, wherein prioritisation of recommendations is conducted by using mathematical pairwise comparisons	Relative weights corresponding to each comparison is ranked and the one with the highest weight is identified as the highest priority

NR, not reported.

### Method development

Three methods were based on existing methods like the bowtie method or the failure mode, effects and criticality analysis methodology.^13 18 21^ Two studies did not describe where the development of the method was based on.^17 19^ Five methods were based on expert opinion,^11 12 14–16^ and in four studies, the experts involved in the development were described.^11 12 14 16^ The number of experts involved ranged from 3 to 57 per study. One study validated the developed method.^11^ More detailed information regarding the development of the methods can be found in table 2. A narrative summary of the methods is presented in online supplemental file 2.

10.1136/bmjoq-2019-000843.supp2 Supplementary data

**Table 2:** Development of the methods for prioritising recommendations

Study	Description of the development of the method	Development of method based on
Brandrud *et al*^11^	The items included in the CPO scale were formulated based on four pillars: the three fundamental questions of the method for improvement (What are we trying to accomplish? How will we know if a change is an improvement? What changes can we make that will result in improvement?),^23^ improvement of literature, final reports of improvement collaboratives of the Norwegian Medical Association and the research team’s discussions	Systematic literature search and expert opinion
Coburn *et al*^12^	An expert panel evaluated the results of a literature review, data analysis from recommended patient safety interventions from national organisations and telephone interview surveys, and began to identify and prioritise a list of rural-relevant patient safety areas and interventions, after which the panel developed the four criteria for evaluating the rural relevance of potential safety interventions	Systematic literature search, interviews and expert opinion
de Dianous and Fiévez^13^	NR	The bowtie method
Flottorp *et al*^14^	The developed checklist was based on desirable attributes selected from existing checklists identified by literature search. The selection of these attributes was built on previous criteria for ‘sensibility’ (the extent to which the criteria are sensible), discussion among collaborators and iterative revisions	Systematic literature search and expert opinion
Geller *et al*^15^	24 behaviour change techniques were distilled from a review of behavioural science literature. The four categories that are hypothesised to have immediate impact on an intervention which are rated by this method are based on literature review and empirical studies of safety belt promotion	Systematic literature search and expert opinion
Hettinger *et al*^16^	Through qualitative analysis of a multi-institutional data set of 334 root cause analysis cases with 782 solutions, a team of safety science experts developed a preliminary model of sustainable and effective solution categories. This model was then modified through interviews of front-line staff regarding selected solutions	Practical experience and expert opinion
McCaughan^17^	NR	NR
McLeod *et al*^18^	NR	Barrier management
Mira *et al*^19^	NR	NR
Rodriguez-Gonzalez *et al*^20^	NR	Failure Mode Effect and Criticality Analysis methodology
Testik *et al*^21^	NR	Cause-and-effect diagrams

NR, not reported.

### Predefined criteria

After completing the data extraction, none of the 11 found methods met all the predefined criteria as shown in table 3.

**Table 3:** Predefined criteria met per method prioritising recommendations

Study	User-friendly	Validation	Recommendations only*	Objective features†
Brandrud *et al*^11^	●	●
Coburn *et al*^12^	●	●
de Dianous and Fiévez^13^	●	●	●
Flottorp *et al*^14^	●	●	
Geller *et al*^15^	●	●	●
Hettinger *et al*^16^	●	●	●
McCaughan^17^	●	●	●
McLeod *et al*^18^	●	●	●
Mira *et al*^19^	●	●
Rodriguez-Gonzalez *et al*^20^	●
Testik *et al*^21^	●

*Recommendations can be prioritised without taking other factors (eg, implementation) into consideration.

†Prioritisation of recommendations is based on objective features (eg, the categories or scores were not affected by personal opinion or knowledge, and the scoring could be expected to be similar when performed by different users).

#### User-friendly

Nine methods were considered user-friendly.^11–19^ The methods described by Rodriguez-Gonzalez *et al* and Testik *et al* were assessed as being not user-friendly.^20 21^ For both methods, comprehensive calculations were necessary, and the method by Testik *et al* cannot be used without extensive mathematical knowledge.

#### Validation

The Change Process and Outcome (CPO) scale was the only validated method.^11^ Validation was performed in multiple stages. For the final 20-item CPO scale, the inter-rater agreement ranged among the six pairs of reviewers from 0.53 (moderate) to 0.75 (strong), median 0.59 (moderate). The test-retest statistic on a sample of four of the single projects was 0.82 (near-complete agreement).

#### Prioritisation of recommendations only

Ten methods were developed to solely prioritise recommendations. The outcome of the CPO scale focussed mainly on the results of the improvement project, and less on the quality of recommendations.^11^

#### Prioritisation based on objective features

Five methods were identified that prioritised recommendations based on objective features, meaning the categories or scores were not affected by personal opinion or knowledge and the scoring could be expected to be similar when performed by different users.^13 15–18^

Although prioritisation of recommendations was not based on objective features, some methods have tried to minimise subjectivity; Flottorp *et al* used a 1 to 5 Likert scale to prioritise recommendations and suggested that at least two people need to assess the recommendations independently and discuss the outcomes afterwards.^14^ Rodriguez-Gonzalez *et al* classified the severity of the potential effect for the patient, the likelihood of occurrence for each failure mode and the likelihood of detecting failure on a 1 to 10 scale. All estimated failure modes were obtained by consensual discussions between team members in order to calculate the risk priority numbers.^20^

## Discussion

Learning and improving healthcare based on the analysis of AEs is a multifactorial process in which every step affects the outcome.^22^ A grading system for recommendations following the AE analysis could have a major impact on the changes for repetition of the AE and on patient safety. It might support prioritisation of the implementation process and clinical effectiveness. The purpose of this review was to identify an appropriate—user-friendly and validated—method that prioritises recommendations following analyses of AEs in healthcare based on objective features. This systematic review identified 11 methods that are all designed to prioritise recommendations. None of the 11 methods met all the predefined criteria. The predefined criteria are essential features to improve and learn from analysing AEs. A broadly used and accepted grading system must be easy to use, objective and preferably validated.

### User-friendly

Out of the 11 methods, nine were user-friendly, meaning they were easy to understand, without time consuming or complex calculations.^11–19^ The methods by Rodriguez-Gonzalez and Testik *et al* were assessed as not being user-friendly as comprehensive calculations were necessary for both methods and the method by Testik *et al* cannot be used without extensive mathematical knowledge.^20 21^

### Validation

The CPO scale was the only validated method in this systematic review.^11^ Validation of a method should identify, and ideally eventually eliminate, the inter-user variability. It is valuable in extrapolation to other centres or fields and will aid in the implementation. For example, the method developed by Geller *et al* has 24 different approaches to change behaviour in a table with scores to calculate the effects.^15^ Furthermore, they also stated an intervention usually consists of a number of behaviour change techniques and therefore the relevant scores need to be added. This results in abundant combinations of scores. All items separately can be considered objective features, but considering the number of possible combinations, the method is prone to significant inter-user variability. Without a validation process of this method, it might be less valuable and difficult to implement. The methods by de Dianous and Fiévez, McCaughan *et al* and McLeod *et al* had a few and clearly defined categories in a well-arranged ordinal sequence.^13 17 18^ Even though these methods were not validated, the scoring system consists of only a few categories based on objective features, which makes these methods less prone to inter-user variability.

### Prioritisation of recommendations only

The CPO scale developed by Brandrud *et al* focussed mainly on the results and quality of the improvement project, and less on the quality of recommendations itself, limiting its use in the prioritisation of recommendations.^11^

### Prioritisation based on objective features

In six methods, the performance of the method was judged to be user-dependent and subjective.^11 12 14 19–21^ For example, some of these methods rated each intervention on a 1 to 5 Likert scale and another assessed understandability, feasibility and usefulness of each recommendation on a 0 to 10 scale.^12 19^ The user is supposed to rate the quality and effectiveness of recommendations using a number or grade based on their own opinion and experience. The outcome of this evaluation may vary significantly depending on the user. For example, a recommendation rated as a 4 out of 10 for user 1, might be rated a 7 out of 10 by user 2 based on their own personal experiences. Therefore, these methods are considered less appropriate to prioritise recommendations in clinical practice and for prioritisation. We believe that prioritising recommendations using categories—for example, of the bowtie method—is more objective than using a ranking by a Likert scale.

Four methods had clear categories.^13 16–18^ Two of these four methods were based on the bowtie method and barrier management.^13 18^ Although this might require a certain knowledge of these existing methods, it is a more objective way of prioritising recommendations. It will result in a more transparent prioritisation. Also, the existing methods are already widely used and have proven to be effective in other industries.

Hettinger *et al* created a method which places each recommendation in one of 13 predefined categories.^16^ Each category was assigned a specific value and was placed in a two-dimensional framework showing the prioritisation of the recommendations. This method might be a promising method for prioritising recommendations as the categories are clear and objective. A drawback of this method is that the values assigned to the categories were assigned in a subjective manner. Through interviews with front-line staff in which the staff was asked to rate each category, the value of each category was determined. Without validation, the method might therefore be less suitable for extrapolation to other circumstances or other settings.

### Methods best suitable for quality improvement in clinical practice

Five methods meet three out of the four predefined criteria and might therefore be considered the best suitable methods for quality improvement in clinical practice.^13 15–18^ However, as stated previously, the ‘Taxonomy of behaviour change strategies to guide intervention development and evaluation’ by Geller *et al* might be more prone to inter-user variability without being validated, and the ‘Model of sustainability and effectiveness in root cause analysis solutions’ described by Hettinger *et al* is less objective than the other methods.^15 16^ Although the ‘Summary of the relationships between components of a barrier system’ by McLeod *et al* has clear categories for the prioritisation of recommendations, these categories must also meet some other criteria, making the method more complicated than the ‘Typology of safety functions’ described by de Dianous and Fiévez, and the ‘Hierarchy of hazard controls’ by McCaughan.^13 17 18^ The methods by de Dianous and Fiévez, and McCaughan have the most potential to select high-quality recommendations. They have few and clearly defined objective categories in a well-arranged ordinal sequence, which makes them user-friendly. Furthermore, the inter-user variability is expected to be limited.

### Limitations

A limitation of this study was the fact there are many different terms that are used for this specific topic. There is little validated research and the lack of MeSH-terms makes it difficult to identify studies in general. Documents and guidelines available to healthcare workers involved in incident analysis, which also address recommendations and which are often only available nationally or even regionally, might not have been retrieved. Even though, our literature search was extensive and in all relevant databases.

No appropriate quality assessment tool was available for this type of research. There are only a few available quality assessment tools for qualitative research. Unfortunately, none of these would be appropriate for all studies included in this review as they are original research papers as well as derived from a guideline and a textbook.^17 18^ In addition, we believe that the quality assessment does not necessarily reflect the quality of the method for prioritising recommendations.

Although successful implementation of recommendations is essential, this was considered beyond the scope of this review. Studies solely regarding implementation were therefore excluded. An appropriate method facilitates selecting high-quality recommendations for implementation. Implementation of high-quality recommendations will probably decrease the number of (similar) AEs, serving the goal of improving the quality of healthcare.

Learning from AEs is only a limited part of improving healthcare as a whole. AEs often result from the daily variations in our processes. Understanding this variation is an important factor in evaluating AEs. A proactive approach and learning from best practices are at least as important. It remains important to evaluate and repair safety gaps in our system if possible. For the repair of these gaps, the completion of the process of learning is essential: reporting AEs, analysing AEs, formulating recommendations, implementing recommendations and evaluating the effect of recommendations.^22^ Underperformance or omitting one of the steps will render the complete process useless.

### Future perspective

The difficulty to translate the knowledge and experience of experts to quantitative data is reflected in the diverse methods used in the different studies. Ideally, there would be a validated, user-friendly method to prioritise recommendations objectively. Combining the experience of healthcare workers and the knowledge of experts specialised in analyses of AEs in other safety-critical industries, holds great potential in creating a solid method that facilitates prioritisation of recommendations in healthcare. A method that would enable users to select the high-quality recommendations for implementation and give insight in factors determining the quality of recommendations following analyses of AEs, would make repetition of AEs in healthcare impossible or less likely.

## Conclusion

There are several methods available to prioritise recommendations following analyses of AEs. All these methods can be used to discuss and select recommendations for implementation. None of the methods is a user-friendly and validated method that prioritises recommendations based on objective features, despite this being an essential feature to improve and learn from analysing AEs. Although there are possibilities to further improve their features, the ‘Typology of safety functions’ by de Dianous and Fiévez, and the ‘Hierarchy of hazard controls’ by McCaughan *et al* have the most potential to select high-quality recommendations as they have only a few clearly defined categories in a well-arranged ordinal sequence.^13 17^ Ultimately, selecting high-quality recommendations for implementation might lead to a decrease in the number of (recurrent) AEs, serving the goal of improving the quality of healthcare.
