# Supplementary material for: Prioritising recommendations following analyses of adverse events in healthcare: a systematic review
Source: BMJ Open Qual. 2020 Oct 9;9(4):e000843. doi: 10.1136/bmjoq-2019-000843 (PMC7549482; doi:10.1136/bmjoq-2019-000843)
Supplement: Supplementary data [file bmjoq-2019-000843supp001.pdf]

Supplementary file 1. Search strategy

Date of search: March 9th 2018

PubMed/MEDLINE

("Professional Practice/standards"[Majr] OR "Primary Health Care/organization and administration"[MAJR] OR primary care[tiab] OR hospital[ti] OR health care[ti] OR "Accident Prevention/standards"[Majr] OR "Accident Prevention/methods"[Majr])

AND

("Checklist"[Majr] OR recommendation*[tiab] OR safety intervention*[tiab] OR remedial action*[tiab] OR safety model*[tiab] OR quality program*[tiab] OR improvement model*[tiab] OR improvement tool*[tiab] OR improvement project*[tiab])

AND

("Quality Improvement"[Mesh] OR "Quality of Health Care"[Mesh:NoExp] OR "Organizational Innovation"[Majr:NoExp] OR "Safety Management/methods"[Majr:NoExp] OR quality improv*[tiab] OR quality management[tiab] OR healthcare quality[tiab] OR safety improvement*[tiab])

AND

("Validation Studies as Topic"[Mesh] OR "Validation Studies" [Publication Type] OR valid*[tiab] OR useful*[tiab] OR usabilit*[tiab])

Embase (Ovid)

#	Searches
1	*health care organization/ or *professional practice/ or *primary health care/ or *accident prevention/ or primary care.ti,ab,kw. or hospital.ti. or health care.ti.
2	*checklist/ or change management/ or (recommendation* or safety intervention* or remedial action* or safety model* or quality program* or improvement model* or improvement tool* or improvement project*).ti,ab,kw.
3	*health care quality/ or *total quality management/ or *safety/ or (quality improv* or quality management or healthcare quality or safety improvement*).ti,ab,kw.
4	validation study/ or (valid* or useful* or usabilit*).ti,ab,kw.
5	1 and 2 and 3 and 4

Cochrane Library

ID Search Hits

#1 MeSH descriptor: [Professional Practice] explode all trees and with qualifier(s): [Standards - ST]

#2 MeSH descriptor: [Primary Health Care] explode all trees and with qualifier(s): [Organization & administration - OG]

#3 MeSH descriptor: [Accident Prevention] explode all trees

#4 primary care:ti,ab,kw (Word variations have been searched)

#5 hospital or health care:ti (Word variations have been searched)

#6 #1 or #2 or #3 or #4 or #5

#7 MeSH descriptor: [Checklist] explode all trees

#8 recommendation* or safety intervention* or remedial action* or safety model* or quality program* or improvement model* or improvement tool* or improvement project*:ti,ab,kw (Word variations have been searched)

#9 #7 or #8

#10 MeSH descriptor: [Quality Improvement] explode all trees

#11 MeSH descriptor: [Quality of Health Care] explode all trees

#12 MeSH descriptor: [Organizational Innovation] explode all trees

#13 MeSH descriptor: [Safety Management] explode all trees

#14 quality improv* or quality management or healthcare quality or safety improvement*:ti,ab,kw (Word variations have been searched)

#15 #10 or #11 or #12 or #13 or #14

#16 MeSH descriptor: [Validation Studies] explode all trees

#17 MeSH descriptor: [Validation Studies as Topic] explode all trees

#18 valid* or useful* or usabilit*:ti,ab,kw (Word variations have been searched)

#19 #16 or #17 or #18

#20 #6 and #9 and #15 and #19

PsycINFO (Ovid)

#	Searches
1	health care delivery/ or health care policy/ or primary health care/ or accident prevention/ or primary care.ti,ab,id. or hospital.ti. or health care.ti.
2	*models/ or (checklist* or recommendation* or safety intervention* or remedial action* or safety model* or quality program* or improvement model* or improvement tool* or improvement project*).ti,ab,id.
3	(quality improv* or quality management or safety management or healthcare quality or safety improvement*).ti,ab,id.
4	1 and 2 and 3

ERIC (Ovid)

#	Searches
1	health services/ or exp primary health care/ or accident prevention/ or primary care.ti,ab,id. or hospital.ti. or health care.ti.
2	check lists/ or (checklist* or recommendation* or safety intervention* or remedial action* or safety model* or quality program* or improvement model* or improvement tool* or improvement project*).ti,ab,id.
3	quality control/ or total quality management/ or safety/ or (quality improv* or quality management or safety management or healthcare quality or safety improvement*).ti,ab,id.
4	1 and 2 and 3
