# Supplementary material for: Prioritising recommendations following analyses of adverse events in healthcare: a systematic review
Source: BMJ Open Qual. 2020 Oct 9;9(4):e000843. doi: 10.1136/bmjoq-2019-000843 (PMC7549482; doi:10.1136/bmjoq-2019-000843)
Supplement: Supplementary data [file bmjoq-2019-000843supp002.pdf]

Supplementary file 2. Narrative summary of the methods

Brandrud *et al* developed the CPO Scale (Change Process and Outcome evaluation instrument) based on a systematic literature search and expert opinion.[11] The scale consists of 20 items and each item is scored with 1 to 5. The results are combined into 3 success levels – *successful*, *promising* and *uncertain* – based on the mean score of items 16 and 19. The projects within each of the 3 success levels are ranked accordingly to the individual sum scores. The CPO Scale is validated.

In Coburn *et al*, after a systematic literature search, interviews and expert opinion, experts rated each intervention using 4 criteria on a 1 to 5 scale.[12] The mean scores are calculated and the interventions with a mean score of 4 or higher on 3 of the 4 criteria are included. Some interventions with lower scores are also included if the experts believe the intervention is important.

The arrangement of de Dianous and Fiévez is based on the bowtie method.[13] They identify four categories. From strongest to weakest barrier the categories are *avoid*, *prevent*, *control* and *limit*, *reduce or mitigate.* They each have a different effect on the occurrence of an unwanted event. For example, an unwanted event can be 'falling down when working at height'. An *avoid* barrier makes the occurrence of the unwanted event impossible. The hazard 'working at height' will be eliminated. No one will be 'working at height', therefore no one can 'fall down when working at height'. A *prevent* barrier puts obstacles before the unwanted event can occur. The hazard 'working at height' still exists. However, a safety belt attached to the person 'working at height' and a fence will *prevent* the person from falling down. A *control* barrier will not stop the unwanted event from occurring. However, it will lead to a safe situation afterwards. A *limit* barrier can reduce the consequences of an unwanted event. The person 'falls down when working at height' and lands on an inflatable cushion to reduce the chance of major injury.

Flottorp *et al* developed a worksheet based on a systematic literature search and expert opinion, upon which every recommendation should be scored 1 to 5 based upon 4 questions.[14] They suggest that at least 2 people should independently assess the recommendations and discuss the results. They do not assign fixed weights to the 3 criteria. However, they do state if a recommendation scores low, the priority is also likely to be low.

Geller *et al* designed a table.[15] All 24 behaviour change techniques, derived from a systematic literature search and expert opinion, are placed on the left side of the table. The 4 following columns are scored with 0 or 1; 0 if the aspect is absent, 1 if the aspect is present. The sum of those points are the *immediate effects* in the fifth column. Then, the *intrinsic control* is scored with 0 (absent) or -1 (present). The *long term effects* are the sum of the *immediate effects* and the *intrinsic control*. Ultimately, this generates a score of 1 to 4 for both the *immediate effects* and the *long term effects.*

Hettinger *et al* created the *model of sustainability and effectiveness in root cause analysis (RCA) solutions*, which is based on practice and expert opinion.[16] A team of experts developed a method of sustainable and effective solution categories based on a multi-institutional dataset of 334 RCA cases and 782 solutions. Through interviews of frontline staff the method was modified based on the 32 most recent RCA cases, from 1 to 5 years after being implemented. The 13 categories are placed on a 2-dimensional framework with the effectiveness along the *y*-axis and the sustainability over time on the *x*-axis. Each scale is graded from minimally to highly effective or sustainable solutions and recommendations can be placed in the categories.

McCaughan uses five categories of control: *elimination*, *substitution*, *engineering controls*, *administrative procedures* and *work practice controls*.[17] *Elimination* being the strongest and *work practice controls* being the weakest control. They state the process should be redesigned and the hazard should be removed. If this is not successful or practical, the next control measure should be used. The last category should only be considered after all the previous measures have been considered and found to be impractical or unsuccessful.

The method of McLeod *et al* is based on the principles for barrier management.[18] It demonstrates the various barrier elements and how they are related to each other. They can be technical, human or rely on a combination of both. Human barrier elements can be organizational or operational. Organisational barriers describe exactly what should be done in rules, instructions or procedures. In operational barriers the responsibility is left to the individuals. They rely on the skill and experience of the individuals involved.

In Mira *et al* experts assessed the *understandability*, *feasibility* and *usefulness* of recommendations using a scale of 1 to 10.[19] A score of 7 or higher is being considered a positive rating.

Based on the failure modes, effect and criticality analysis (FMECA) methodology, the *severity* of the potential effect, the likelihood of *occurrence* and *detecting* are classified on a scale of 1 to 10 through consensus between team members in the study of Rodriguez-Gonzalez *et al*.[20] The product of these three numbers is the risk priority number (RPN). The maximum RPN is 1000 and at least one improvement is determined when the RPN is higher than 100. A priority classification is established by taking into consideration the value of the criticality index, the extent of the expected reduction in criticality and the volume of work and expenditure needed to develop the proposal. Ultimately, the recommendations are prioritized per time limit, 12 months to over 48 months.

The experts in the Testik *et al* study construct a pairwise comparison matrix **A** with comparison values based on a 1 to 9 scale.[21] Then a normalized comparison matrix **N** is obtained with relative weights **w**. λ_{max} is obtained of matrix **A** and used in evaluating the consistency of the pairwise comparisons through consistency index (CI = $(\lambda_{max} – n) / (n – 1)$). 4. CI is compared with the random index (RI) to obtain the consistency ratio (CR = CI / RI). A CI of 0 indicates a perfectly consistent matrix, although slight inconsistencies are tolerated up to a CR of 0.1. The relative weights **w** corresponding to each comparison is ranked and the one with the highest weight is identified as the highest priority.
